# Combination Therapy with 1% Nanocurcumin Gel and 0.1% Triamcinolone Acetonide Mouth Rinse for Oral Lichen Planus: A Randomized Double-Blind Placebo Controlled Clinical Trial

**DOI:** 10.1155/2020/4298193

**Published:** 2020-05-20

**Authors:** Mahin Bakhshi, Shahzad Gholami, Arash Mahboubi, Mahmoud Reza Jaafari, Mahshid Namdari

**Affiliations:** ^1^Department of Oral Medicine, School of Dentistry, Shahid Beheshti University of Medical Sciences, Tehran, Iran; ^2^Food Safety Research Center, Department of Pharmaceutics, School of Pharmacy, Shahid Beheshti University of Medical Sciences, Tehran, Iran; ^3^Department of Pharmaceutical Nanotechnology, School of Pharmacy, Mashhad University of Medical Sciences, Mashhad, Iran; ^4^Department of Community Oral Health, School of Dentistry, Shahid Beheshti University of Medical Sciences, Tehran, Iran; ^5^Department of Biostatistics, School of Allied Medical Sciences, Shahid Beheshti University of Medical Sciences, Tehran, Iran

## Abstract

**Objectives:**

This study aimed to evaluate the efficacy of a combination of 1% nanocurcumin gel with 0.1% triamcinolone acetonide mouth rinse for oral lichen planus (OLP).

**Materials and Methods:**

This double-blind randomized clinical trial was conducted on 31 patients with erosive or ulcerative OLP. All patients received 0.1% triamcinolone mouth rinse and were then randomly divided into two groups for combination therapy with (I) %1 nanocurcumin gel or (II) placebo gel. The reticular-erosive-ulcerative (REU) score was calculated at baseline and at two and four weeks after the intervention. The changes in the mean REU score and the efficacy index were calculated to determine the level of improvement after two and four weeks. Data were analyzed using independent *t*-test, repeated measures ANCOVA, Mann–Whitney test, and chi-square test. *P* < 0.05 was considered statistically significant.

**Results:**

There were 14 patients in the nanocurcumin and 17 patients in the placebo group. A significantly higher decrease in the mean REU score was observed in the nanocurcumin compared with the placebo group (*P* < 0.001). The efficacy index was significantly higher in the nanocurcumin group (*P* < 0.001).

**Conclusion:**

Application of 1% nanocurcumin in combination with 0.1% triamcinolone acetonide can serve as an effective treatment strategy to enhance the level of improvement of lesions compared with the use of triamcinolone acetonide alone.

## 1. Introduction

Oral lichen planus (OLP) is a chronic mucocutaneous disorder with an unknown etiology [1–4]. It occurs as a result of an immunological cytotoxic reaction against keratinocytes, causing vacuolar degeneration of basal cells [5]. The clinical manifestation of OLP includes a white patch with red components in reticular, papular, plaque-like, vesicular, erythematous, erosive, or ulcerative forms [4]. The diagnosis and treatment of erosive, atrophic, and ulcerative types are clinically important due to having symptoms such as burning sensation and pain as well as the potential for malignant transformation [6]. Treatment of OLP aims to decrease erythema, resolve the mucosal ulcers, and decrease the disease symptoms during the course of activation and prolong the remission period. Corticosteroids are the basis of treatment of symptomatic OLP [4]. However, due to the chronic nature of the disease, it requires long-term corticosteroid therapy, which can have numerous complications and side effects such as candidiasis, burning sensation, and bad taste in the mouth, mucosal atrophy, nausea, sore throat, and xerostomia. Also, long-term systemic corticosteroid therapy may even cause adrenal insufficiency [4]. Triamcinolone acetonide is a moderate-to-strong corticosteroid supplied in the form of an ointment and 0.1% mouth rinse. It is the most commonly used medication for treatment of mucosal lesions due to its lower rate of complications compared with other medications [7]. However, some patients may require its long-term use or replacement with stronger steroids in case of resistance of lesions to this medication [8]. In such cases, using triamcinolone acetonide in combination with an herbal medication to enhance its efficacy can be helpful for treatment of OLP lesions. Among herbal medications, turmeric is commonly used in nutritional regimens. Its effective substance is curcumin, which is a polyphenol, rich in strong antioxidants comparable with vitamin *C* and vitamin *E*. It eliminates the reactive oxygen species including superoxide amine and hydroxyl radicals. It also has antifungal effects and can prevent candidiasis, which is a common complication of using corticosteroids [9–11].

However, the clinical use of curcumin has some shortcomings including its low solubility, high rate of metabolism, and low bioavailability. Several strategies have been suggested to overcome these limitations including the use of nanosystems [12]. Nanocurcumin may be used to overcome the low solubility of curcumin, and nanoformulation of curcumin obviously enhances its effectiveness and bioavailability in vitro and in vivo [13]. It can easily pass through the biological barriers, increase the interaction with host cells, and eliminate the microbial agents. Nanocurcumin also increases the formation of granulation tissue and angiogenesis [14]. Oral and topical use of curcumin have shown successful results in treatment of OLP. Evidence shows that oral intake of curcumin decreases the level of pain and erythema in OLP; however, it is associated with complications such as gastrointestinal problems [15]. Considering the fact that curcumin is known to be effective for treatment of OLP, and lack of studies on the efficacy of nanocurcumin combined with 0.1% triamcinolone acetonide for treatment of OLP, this study aimed to assess the effect of combined use of 1% nanomicelle curcumin gel and 0.1% triamcinolone acetonide mouth rinse for treatment of OLP.

## 2. Patients and Methods

### 2.1. Study Design

This randomized, double-blind, placebo-controlled clinical trial compared the efficacy of combination therapy with 1% nanocurcumin gel and 0.1% triamcinolone acetonide mouth rinse in group I and placebo gel plus 0.1% triamcinolone acetonide mouth rinse in group II for treatment of OLP. Patients were evaluated at baseline, and two more visits were scheduled at two and four weeks after treatment.

The study protocol was thoroughly explained to patients prior to the study and their written informed consent was obtained. The study was approved by the ethics research committee of Dental Faculty of Shahid Beheshti University of Medical Sciences (IR.SBMU.DRC.REC.1397.014) and registered in the Iranian Registry of Clinical Trials (IRCT20190523043678N1).

### 2.2. Participants

The patients with symptomatic (erosive and ulcerative) OLP who complained of pain and/or burning sensation participated in this study. All patients who met the clinical criterion for diagnosis of OLP (Wickham striae) or had histopathologically confirmed OLP (in suspected cases) were enrolled [16]. Patients were selected among those presenting to the Oral Medicine Department. The inclusion criteria were absence of topical, local, or systemic corticosteroid therapy during the past one month [17] and no use of analgesics or anesthetic agents. The exclusion criteria were lichenoid reactions due to medication intake or dental materials, pregnancy [18], history of malignancy [19], noncooperative patients, and patients who did not correctly follow the instructions on using the medications [20].

### 2.3. Sample Size

The number of patients in each group was calculated according to a study by Piboonniyom et al. [21] assuming that the new treatment modality would cause a reduction in the reticular-erosive-ulcerative (REU) score by averagely 4.5 units compared with the conventional treatment. The standard deviation of reduction in REU score was considered to be 4.2 according to that study too. Therefore, we calculated the number of sample size to be 14 patients in each group, using the following formula with *α* = 5% and study power of 80%: *n*_1_=*n*_2_=(*z*_1−(*α*/2)_+*z*_1−*β*_)^2^2*SD*^2^/(*δ*)^2^.

Considering 15% loss to follow-up, 17 patients were included in each group. The participants were selected by convenience sampling.

### 2.4. Randomization and Allocation

Patients who met the inclusion criteria were randomly assigned to the treatment groups. A random permuted block method was used for this purpose. The process of randomization and the sequence of random allocation of patients to the treatment groups were designed by the Excel RAND function.

The main researcher who was responsible for implementing the treatment and evaluating the results was blinded to the allocation sequence of patients (allocation concealment).

### 2.5. Blinding

Since we did not mean to deprive any patient from the standard treatment of OLP, both groups received triamcinolone acetonide mouthwash. The patients were blinded to the type of gel (1% nanomicelle curcumin gel or placebo gel) which was provided to them in combination with the triamcinolone acetonide mouthwash. The curcumin and placebo gels were supplied in identical tubes of the same shape and color.

The allocation of gels was performed by the second researcher who was not involved in patient evaluation. Neither the main researcher nor the patients were aware of the type of gel administered (1% nanomicelle curcumin gel or the placebo gel).

### 2.6. Intervention

All patients were randomly assigned to the two groups for use of 0.1% triamcinolone mouth rinse combined with %1 nanocurcumin gel or the placebo gel. Since all patients received triamcinolone acetonide, the possible difference in the results could be attributed to the type of gel with high level of certainty. The triamcinolone acetonide mouth rinse was prepared in the School of Pharmacy under the supervision of a pharmaceutical specialist. The 1% nanomicelle curcumin gel was purchased from the market (Sina Darou). The patients were instructed to gargle the triamcinolone mouth rinse three times a day (after each meal) for one minute on a daily basis. They were instructed to apply the gel on the lesions after each time of using the mouth rinse and refrain from eating and drinking for 30 min after use. They were asked to follow this protocol for one month.

### 2.7. Clinical Assessments

Oral lesions were evaluated using a dental mirror and a wooden tongue depressor under a unit light. For clinical examination of OLP lesions, the oral cavity was hypothetically divided into 10 areas [20]: (I) upper and lower lips, (II) right buccal mucosa, (III) left buccal mucosa, (IV) dorsum of the tongue, (V) ventral surface of the tongue, (VI) floor of the oral cavity, (VII) hard palate, (VIII) soft palate and tonsils, (IX) maxillary gingiva, and (X) mandibular gingiva. Next, the REU clinical score was determined based on the presence of reticular areas, size of erythema, and size of wound [21]. “R” indicates the presence of reticular/papular areas in this scoring system. In case of presence of such areas, score 1 is allocated to this parameter, and in case of their absence, score 0 is allocated (score 0: absence of white lines; score 1: presence of white lines or a keratotic papule). “E” and “U” indicate the presence of erosive and erythematous areas, respectively (0: absence of lesion; 1: lesions smaller than 1 cm^2^; 2: lesions between 1 and 3 cm^2^; 3: lesions larger than 3 cm^2^). The lesions were scored from 0 to 3 accordingly. Thus, one score was allocated to each of the three clinical symptoms in all 10 areas. The clinical score of each patient was calculated as the sum of score of reticular and sum of score of erythematous areas multiplied by 1.5 plus the sum of score of erosive areas multiplied by 2 [22].

### 2.8. Outcome Measurements

The patients were clinically examined two and four weeks after using the medications, and the REU clinical score was calculated again for them to determine the severity of the lesions. The calculated scores for each patient were recorded at two and four weeks. The efficacy index of clinical REU improvement was calculated using the formula *EI* = [*(V15 or V30* – *V1)* ÷ *V1*] × *100%* where V15 and V30 refer to the values measured at two and four weeks, respectively, while V1 is the baseline REU clinical score [23].

The efficacy index was classified as follows to determine the level of improvement [23, 24]:

1. Complete healing: efficacy index of 100%
2. Marked improvement: 70%< efficacy index <100%
3. Moderate improvement: 30%< efficacy index <70%
4. No improvement: efficacy index <30%

### 2.9. Statistical Analyses

Independent *t*-test was applied to compare the mean REU of the two groups at baseline. Since the baseline values were not completely comparable between the two groups, they were considered as covariates, and repeated measures ANCOVA was applied for the comparison of REU scores at two and four weeks. The adjusted mean values were then compared. The Bonferroni adjustment was used for multiple comparisons. Fisher's exact test and Mann–Whitney test was used as well. Statistical analyses were performed using SPSS version 21 (IBM Corp., Armonk, NY, USA).

## 3. Results

### 3.1. Characteristics of the Participants

A total of 31 patients including seven males (22.6%) and 24 females (77.4%) precipitated in this study. Of all, 14 patients were evaluated in the nanocurcumin (triamcinolone plus nanocurcumin gel) and 17 patients in the placebo group (triamcinolone plus the placebo gel).

There were three (21.4%) and four (23.5%) males in the nanocurcumin and placebo groups, respectively. No significant difference was found between the groups regarding gender (*P*=1). The mean (standard deviation) age of patients was 59 (15.12) years (range 42 to 85 years) in the nanocurcumin and 48 (12.71) years (range 25 to 67 years) in the placebo group. The difference in the mean age of patients was not significant between the two groups (*P*=0.34).

In terms of location of OLP lesions, the most common site of involvement was the buccal mucosa in both groups.  Table 1 presents the location of OLP lesions in the two groups.

### 3.2. Outcomes

Due to the slightly higher mean REU score in the nanocurcumin group, compared with the placebo at baseline, the baseline REU values were taken as covariates, and repeated measures ANCOVA was applied and the adjusted mean REU values were then compared. Subsequent comparison of the adjusted mean values showed significantly higher REU score in the placebo group at two and four weeks after the onset of treatment (*P* < 0.001 and *P* < 0.001, respectively). Therefore, significantly superior results were obtained in the nanocurcumin compared with the placebo group at the follow-up sessions. The details for the REU values are presented in Table 2.

**Figure 1:**
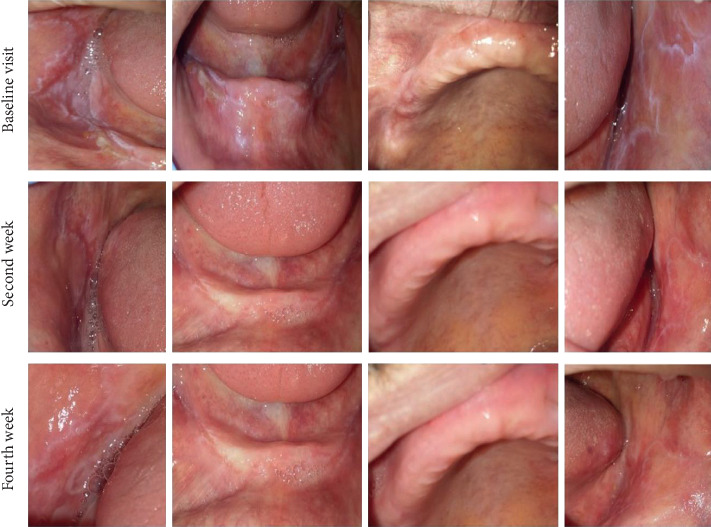
Intraoral photographs of a patient in the nanocurcumin group at baseline and at two and four weeks.

**Figure 2:**
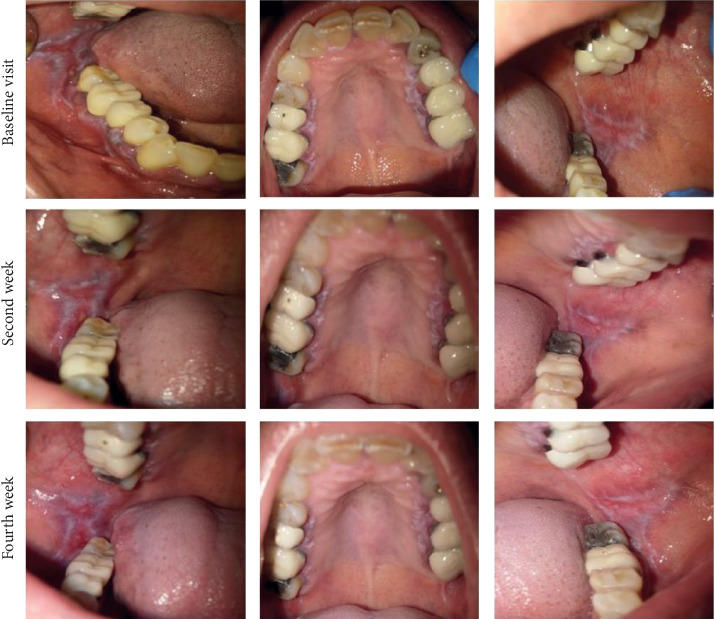
Intraoral photographs of a patient in the placebo group at baseline and at two and four weeks.

Figures 1 and 2 show intraoral photographs of patients in the nanocurcumin and placebo groups at baseline and after two and four weeks.

At both two and four weeks, significant differences were noted in the efficacy index categories between the two groups (*P* < 0.001 and *P*=0.001, respectively).  Table 3 shows the distribution of the efficacy index categories in the two groups at two and four weeks. Complete healing was not seen in any group. However, number of patients in the placebo group who showed no improvement was significantly higher than the nanocurcumin group.

## 4. Discussion

This study assessed the effect of combined use of 1% nanomicelle curcumin gel and 0.1% triamcinolone acetonide mouth rinse in comparison with 0.1% triamcinolone acetonide alone for treatment of OLP. OLP is an immunity-mediated inflammatory chronic disease that mainly involves the oral mucosa. Some of its subtypes, such as the reticular type, do not require a specific treatment. However, its erosive and ulcerative types are associated with pain and discomfort and decrease the quality of life of patients and have a risk of malignant transformation. Thus, they require treatment [6]. The current results revealed that combined use of 1% nanomicelle curcumin gel plus 0.1% triamcinolone mouth rinse was significantly more effective than the use of triamcinolone mouth rinse alone for treatment of OLP and significantly decreased the severity of clinical symptoms and enhanced the rate of improvement. In the present study, the REU clinical score was used to determine the severity of OLP lesions [21]. This scoring system is easy to use, and its results are comparable to those of NRS. It can be effectively used to assess the rate of improvement [25]. In our study, this score was 20.39 ± 8.54 at baseline, which decreased to 11.04 ± 7.32 and 7.21 ± 5.42 at two and four weeks, respectively, in the nanocurcumin group. This reduction was significantly greater than that in the placebo group (*P* < 0.001). In other words, nanocurcumin had a higher efficacy in decreasing the extent and type of oral lesions (ulcer and erythema). The clinical score decreased in both groups at two and four weeks compared with baseline. Keshari et al. in their clinical trial in 2015 compared the efficacy of topical nanocurcumin with triamcinolone for improvement of OLP. They assessed the patients at seven and 15 days and found that nanocurcumin was more effective than triamcinolone for resolution of erythema but had a lower effect on ulcers. In the present study, the clinical score was determined as the sum of scores for ulcers and erythema but in the study by Keshari et al. erythema and ulcers were separately scored because they used the modified oral mucositis index, which is used for assessment of the mucositis lesions. In the clinical setting, differentiation between ulcer and erythema in OLP (to assess the overall effect of medications on the severity of clinical symptoms) is difficult and challenging. Thus, a simpler clinical scale is always preferred for this purpose [18]. Chainani et al. in 2007 evaluated the effect of daily intake of 2000 mg curcumin on OLP and concluded that this dosage of curcumin had no significant effect on disease symptoms [26]. Another study in 2012 evaluated the effect of oral intake of 6000 mg curcumin daily and used the NRS for erythema and ulcers to assess the course of healing. They showed that the severity of symptoms significantly decreased after two weeks in the curcumin group. They indicated that increasing the dosage of oral intake of curcumin resulted in significant improvement of OLP symptoms. However, it had side effects such as gastrointestinal problems in many patients. Comparison of their results and ours indicates the superiority of topical use of curcumin compared with its systemic administration. Also, it should be noted that the placebo group did not receive any treatment in their study [27]. Kia et al. in 2017 evaluated the effect of oral intake of nanocurcumin on clinical manifestations of OLP using the Thongprasom index. Of the 10 patients that were evaluated in their study, 50% experienced pain reduction and 80% experienced improvement in their clinical symptoms. Their results, in line with our findings, highlighted the advantage of using nanocurcumin for treatment of OLP. However, the Thongprasom index does not address the location of lesions in cases with multiple lesions; however, this problem can be resolved by using the REU clinical score [28]. In their study, patients who used oral nanocurcumin for four weeks well tolerated it and reported no side effects, which highlights the superiority of oral intake of nanocurcumin compared with curcumin. This finding supports the use of nanocurcumin in the present study. Another advantage of the present study was calculation of the efficacy index. Accordingly, the percentage of primary improvement in nanocurcumin and placebo groups was 54.60 ± 17.05 and 24.63 ± 11.71, respectively. The percentage of secondary improvement in nanocurcumin and placebo groups was 63.04 ± 13.94 and 36.46 ± 17.53, respectively. The difference in this respect was significant between the two groups such that the percentage of primary and secondary improvement in the nanocurcumin group was significantly higher than that in the placebo group, and curcumin significantly enhanced the healing of OLP lesions.

In a systematic review in 2019, Lv et al. evaluated the clinical efficacy of curcumin for treatment of OLP. They reviewed nine articles including six clinical trials, two pilot studies, and one case report. Seven of the reviewed articles showed significant reduction of pain and clinical symptoms of lesions after treatment with curcumin compared with baseline. Three studies compared the efficacy of curcumin and corticosteroids. They found no significant difference between the two groups [29]. Keshari and Chainan used the NRS for assessment of the level of pain [18, 27]. We did not use this scale to determine the level of pain in our study because we believe that assessment of the efficacy of medications based on clinical observations can be more accurate compared with patient-reported symptoms. On the other hand, according to Park et al. in 2012, a direct and parallel correlation exists between the REU clinical score and NRS such that patients with decreased erythema and ulcer often experience lower level of pain and discomfort [25]. Similar to our study, Keshari and Chainan showed the positive efficacy of curcumin. However, they did not assess the rate of improvement [18, 27].

Thomas et al. in 2017 compared the efficacy of 1% curcumin gel and topical triamcinolone acetonide in OLP patients. Patients in group one used triamcinolone ointment while patients in groups two and three used curcumin gel three times and six times daily, respectively, for three months. They showed that the level of pain and the severity of clinical symptoms decreased in all three groups at three months compared with baseline. Comparison of the three groups revealed that application of 1% curcumin gel six times a day decreased pain by 67% and mucosal burning by 77%. These effects were comparable to those of triamcinolone acetonide. They suggested that curcumin can be used as a medication in the maintenance phase of treatment of OLP because long-term corticosteroid therapy can have many side effects [19]. Application of curcumin six times a day may be difficult for patients; thus, it can be used along with a corticosteroid in the initial phase of treatment. The current study was designed to assess the efficacy of combined use of corticosteroid and curcumin for treatment of OLP.

Kia et al. in 2015 compared the efficacy of topical use of 5% curcumin and 0.1% triamcinolone acetonide for treatment of OLP. They showed that both treatments were equally effective. The difference between our study and theirs was the concentration of curcumin since they used 5% concentration of curcumin. However, in the present study, nanocurcumin was used in a lower dosage which is an advantage in comparison with the use of curcumin in a higher concentration for treatment of OLP [17].

In the current study, the nanocurcumin gel was not available as a mucosal patch; thus, its substantivity in the oral cavity was lower compared with the use of a mucosal patch. Future studies are required to produce nanocurcumin mucosal patches to increase its substantivity in the oral environment.

Use of herbal medications in combination with steroids is always preferred to the combined use of two chemical medications. Corticosteroids modulate the immune response and inflammation. Similarly, curcumin can modulate the immune response by activating the macrophages and natural killer cells and modulating the activity of lymphocytes. Thus, it enhances the efficacy of corticosteroids as well. Triamcinolone acetonide was used in both groups in our study since we did not want to deprive any patient from the standard treatment of OLP. In the test group, we also used nanocurcumin, which is a low-risk herbal medication to enhance the rate of improvement (considering the low dose and the need for long-term use of triamcinolone). This was done to enhance the efficacy of triamcinolone and decrease the need for strong corticosteroids. Also, considering the risk of malignant transformation of erosive and ulcerative OLP lesions [6], it would be ideal to use a safe anticancer medication in combination with the standard basic treatment of OLP.

## Figures and Tables

**Table 1:** The distribution of patients according to the location of lesion in two groups.

Site of involvement	Nanocurcumin *n* (%)^*∗*^	Placebo *n* (%)	Total *n* (%)

Buccal mucosa (right)	12 (86)	12 (71)	24 (77)
Buccal mucosa (center)	12 (86)	11 (65)	23 (74)
Maxillary gingiva	12 (86)	11 (65)	23 (74)
Mandibular gingiva	12 (86)	10 (59)	22 (71)
Lip (upper)	2 (14)	2 (12)	4 (13)
Lip (lower)	2 (14)	2 (12)	4 (13)
Floor of the mouth	2 (14)	0 (0)	2 (6)
Hard palate mucosa	5 (36)	6 (35)	11 (35)
Tongue (ventral)	4 (29)	3 (18)	7 (23)
Tongue (dorsal)	6 (43)	6 (35)	12 (39)

^*∗*^Values are reported as number (percent).

**Table 2:** Comparison of the REU scores at baseline and at two and four weeks.

Time	Observed mean REU		Adjusted mean REU^*∗*^		Adjusted mean difference	
	Nanocurcumin	Placebo	Nanocurcumin	Placebo	Mean (SE)	*P* value
	Mean (SD)	Mean (SD)	Mean (SE)	Mean (SE)		
Baseline	20.39 (8.54)	16.00 (8.48)				0.163
Two weeks	11.04 (7.32)	12.26 (7.09)	9.09 (0.62)	13.86 (0.54)	−4.77 (0.83)	<0.001^$^
Four weeks	7.21 (5.42)	10.44 (6.51)	5.71 (0.81)	11.67 (0.74)	−5.93 (1.12)	<0.001^$^

^*∗*^Baseline REU values are taken as covariates for the adjusted model. ^$^*P* values are based on comparing the adjusted mean values.

**Table 3:** Comparison of the improvement level and the efficacy index at two and four weeks between the two groups.

	Two weeks			Four weeks		
	Nanocurcumin n(%)	Placebo *n* (%)	*P* value	Nanocurcumin *n* (%)	Placebo *n* (%)	*P* value
Healed	0 (0%)	0 (0%)	<0.001	0 (0%)	0 (0%)	0.001
Marked improvement	2 (14.3%)	0 (0%)		7 (50%)	1 (5.9%)	
Moderate improvement	11 (78.6%)	3 (17.6%)		7 (50%)	9 (52..9%)	
No improvement	1 (7.1%)	14 (82.4%)		0 (0%)	7 (41.2%)	

EI mean (SD)	54.60 (17.05)	24.63 (11.71)	<0.001	63.04 (13.94)	36.46(17.53)	<0.001

## Data Availability

The data used to support the findings of this study are available from the corresponding author upon request.
